# Liquified Petroleum Gas (LPG) Supply and Demand for Cooking in Northern Ghana

**DOI:** 10.1007/s10393-018-1351-4

**Published:** 2018-08-14

**Authors:** Maxwell Dalaba, Rex Alirigia, Elise Mesenbring, Evan Coffey, Zachary Brown, Michael Hannigan, Christine Wiedinmyer, Abraham Oduro, Katherine L. Dickinson

**Affiliations:** 1 Navrongo Health Research Centre, Navrongo, Ghana; 2 University of Colorado Boulder, Boulder, CO USA; 3 North Carolina State University, Raleigh, NC USA; 4 Colorado School of Public Health, University of Colorado Anschutz, 13001 E 17th Pl., Campus Box B119, Aurora, CO 80045 USA

**Keywords:** Cookstoves, Liquefied petroleum gas (LPG), Technology adoption, Household energy, Fuel supply, Behavior change

## Abstract

Like many other countries, Ghana relies on biomass (mainly wood and charcoal) for most of its cooking needs. A national action plan aims to expand liquefied petroleum gas (LPG) access to 50% of the country’s population by 2020. While the country’s southern urban areas have made progress toward this goal, LPG use for cooking remains low in the north. The aim of this cross-sectional study was to characterize the current state of the LPG market in this area and examine opportunities and barriers to scale up LPG adoption. We interviewed 16 LPG suppliers (stove, cylinder, and fuel vendors) as well as 592 households in the Kassena-Nankana Districts (KND) of Ghana. We find large rural–urban differences in LPG uptake: less than 10% of rural households own LPG stoves compared with over half of urban households. Awareness of LPG is high across the region, but accessibility of fuel supply is highly limited, with just one refilling station located in the KND. Affordability is perceived as the main barrier to LPG adoption, and acceptability is also limited by widespread concerns about the safety of cooking with LPG. Transitioning to a cylinder recirculation model, and providing more targeted subsidies and credit options, should be explored to expand access to cleaner cooking in this region.

## Background

In Northern Ghana, the majority of households continue to use biomass (wood or charcoal) to meet their cooking needs. This practice has well-documented impacts on health outcomes and environmental quality, and a national and global push is underway to enable transitions toward cleaner cooking. Liquefied petroleum gas (LPG) is widely advocated as one of the cleanest options to achieve both health and climate change mitigation goals (Rosenthal et al. 2018).

At the national level, Ghana aims to expand LPG access to 50% of the country’s population by 2020 (Ghana Energy Commission 2012). Between 2005 and 2013, the share of households cooking with modern fuels such as LPG and electricity increased from 10.8 to 23.6% (Mensah and Adu 2015). However, most of this progress occurred in urban areas. As of 2014, only 5.5% of rural households used LPG as their main cooking fuel (Ghana Statistical Service 2014). This rural–urban disparity has inspired national policies to expand LPG access and use, most notably through the Rural LPG Promotion Program (RLP) (Modern Ghana 2014).

Exemplifying Ghana’s LPG access challenges, the Kassena-Nankana Districts (KND) are located in Ghana’s Upper East region along the country’s northern border, in one of the country’s poorest regions (Ghana Statistical Service 2014). According to 2011–2013 data, only 7% of households in these districts used LPG as their main cooking fuel, while 74% relied on fuelwood or crop residue and 18% mainly used charcoal (Oduro et al. 2012). Use of LPG was concentrated in the central urban areas around Navrongo town, where about a third of households reported LPG as their main fuel, while 60% relied primarily on charcoal. Outside of the central area, only 3% of the population used LPG as their main fuel.

There is limited knowledge about why LPG usage is low in Northern Ghana. Furthermore, studies on cleaner cooking have focused on household-level adoption decisions and, to a lesser extent, national policies, with less attention to local-level supply-side factors. This paper aims to fill this gap by examining both supply- and demand-side factors affecting LPG adoption in the KND.

### Liquefied Petroleum Gas (LPG) Distribution System in Ghana

In the current distribution model for LPG throughout Ghana, customers purchase LPG stoves and cylinders from retail shops and then refill cylinders at filling stations. International guidelines recommend cylinder recirculation, in which marketers purchase cylinders, and customers pay a deposit and exchange empty cylinders for full ones at dispersed distribution locations (Ghana Energy Commission 2012; Evans et al. 2013). Cylinder recirculation can avoid some safety problems relating to the use of old cylinders since households do not have an incentive to continue using older cylinders. Shortly after an explosion at a natural gas station in Accra in October 2017, the President of Ghana declared that cylinder recirculation would be implemented throughout the country within a year (Adogla-Bessa 2017). Implementation of this policy has previously been delayed by opposition from the LPG Marketers Association and the Association of Gas Tanker Drivers. However, the recent explosion may provide momentum toward this policy. To our knowledge, LPG cylinder recirculation has not yet been tested in Northern Ghana.

## Methods

### Study Site

Our study area is the Kassena-Nankana East and West Districts of the Upper East region of Ghana, collectively referred to by their former name—the Kassena-Nankana District (KND). The KND has a population of 156,000 (~ 20% urban) and covers 1674 km^2^ of land with a hot and arid climate and one rainy season lasting from May to October. The majority of the land is used in subsistence agriculture (Oduro et al. 2012).

### Study Context

The work presented here was conducted as part of ongoing cookstove-focused projects in the KND. In 2013, Research on Emissions, Air quality, Climate, and Cooking Technologies in Northern Ghana (REACCTING) was launched to examine impacts of improved biomass stoves through a randomized intervention in the rural areas of the KND (Dickinson et al. 2015). Building on these efforts, the Prices, Peers, and Perceptions (P3) study began in 2015 to further explore technology adoption through two sets of experiments. The P3 Bio intervention builds directly on the REACCTING study by selling improved biomass stoves to rural households, while the P3 Gas arm is assessing adoption of LPG stoves in the urban areas around Navrongo town.

As part of the first phase of the P3 Gas project, we interviewed local LPG suppliers (*n* = 16) in June–July 2016. Additionally, a baseline household survey covering the P3 Bio and P3 Gas subgroups (*n* = 592) was conducted between December 2016 and February 2017, before either of the interventions began. This paper uses these two data sources to explore LPG supply and demand in the region.

### LPG Supply Interviews

The LPG supplier interviews aimed to characterize the supply chain for LPG stoves, cylinders, and fuel in the KND and identify opportunities and barriers for scaling up this supply chain. The target population was all LPG suppliers servicing this region. We interviewed all retail centers selling LPG stoves or cylinders in the KND. Only one LPG filling station was located in the districts, while additional filling stations were located just outside the KND near the town of Bolgatanga. Since households in the KND often travel to Bolgatanga and may get fuel from these filling stations, and because we wanted to get multiple perspectives from filling stations in this region, we decided to interview all station operators in the larger Upper East region (encompassing the KND).

We developed the supply survey using a literature review (e.g., Lewis and Pattanayak 2012; Cecelski and Matinga 2014; Mensah and Adu 2015) and input from international experts in the household energy sector (e.g., Global LPG Partnership, NIH Clean Cooking Implementation Science Network). Data collected included: types of products sold; problems with the supply of products; perceptions of policies to increase LPG adoption; and potential for shifting to cylinder recirculation.

### Household Demand Surveys

We recruited 300 households each for the P3 Bio and P3 Gas baseline surveys (600 households in total). The P3 Bio recruitment targeted 150 households that were nearest neighbors (peers) of a household that participated in the REACCTING study and 150 households randomly selected from clusters that were not part of that study (non-peers). For the P3 Gas study, we randomly sampled 300 households in the central area of the KND, stratified by distance from the market center/Navrongo town. Random sampling methods for both groups generated samples that are representative of the groups from which they were drawn: rural, primarily biomass-using households for P3 Bio, and urban households for P3 Gas. All urban households are within 5 km of Navrongo town; rural households are more than 5 km from Navrongo town. Eligibility for both groups was limited to households with at least one woman aged 18–55 and one child under five, since these are groups expected to be most vulnerable to cooking emissions and associated health impacts. In each household, we interviewed the primary cook (typically female) and, if present, another individual in the household responsible for making financial decisions (typically male). The survey included questions assessing current cooking practices, including stove stacking (use of multiple types of stoves) and fuel stacking (the use of multiple types of fuel) as well as perceived barriers to LPG adoption.

Both the LPG supply interviews and the household surveys were conducted face to face using Android tablets and the Open Data Kit (ODK) survey software by graduate-level interviewers in either English or one of the local languages of the district. Prices of products were collected in local currency, Ghana cedis (GHC); we convert all prices to USD using the average exchange rate for the study periods: 1USD = GHC4.4.

### Data Analysis

Data were analyzed using STATA (version 15.1), and results are presented in tables and graphs. A linear probability model regression was conducted to assess factors associated with LPG ownership among urban households. (This analysis was not conducted for rural households given that there were very few LPG owners in rural areas.) This regression took the form:

$$ Y_{i} =\upalpha + \varvec{X}_{\varvec{i}} \varvec{\overset{\lower0.5em\hbox{$\smash{\scriptscriptstyle\rightharpoonup}$}} {\beta } } +\upvarepsilon_{i} $$

where $Y_{i}$ was a binary indicator for whether or not the household had an LPG stove, and $\varvec{X}_{\varvec{i}}$ was a vector of household characteristics, including age, gender, and education of the primary cook, whether or not the primary cook was the financial decision maker for the household, whether the household did any farming, household size, access to electricity, financial assets, distance to the central market, and whether or not most of the household’s neighbors had LPG. We present regression coefficients and their 95% confidence intervals graphically using Stata’s *coefplot* command (Jann 2014). This approach takes a step away from interpretation based on “bright lines” of statistical significance (see, for example, Ziliak and McCloskey 2008) and allows an easy visual assessment of coefficient sizes and precision, which are our main interests here.

For several variables, including stove preference and demand indictors, we also examined differences between rural and urban respondents, and between primary cooks and financial decision makers, using Chi-squared tests.

### Ethical Considerations

Ethical approval was obtained from the institutional review boards of the participating institutions (details removed for double-blind reviewing). Community entry meetings were held with local chiefs and subchiefs to explain the objectives of the study and enlist their support in carrying out the study. In addition, all the respondents were briefed about the study procedure and written informed consent was obtained.

## Results

### Spatial Distribution of LPG Supply and Demand

**Figure 1:**
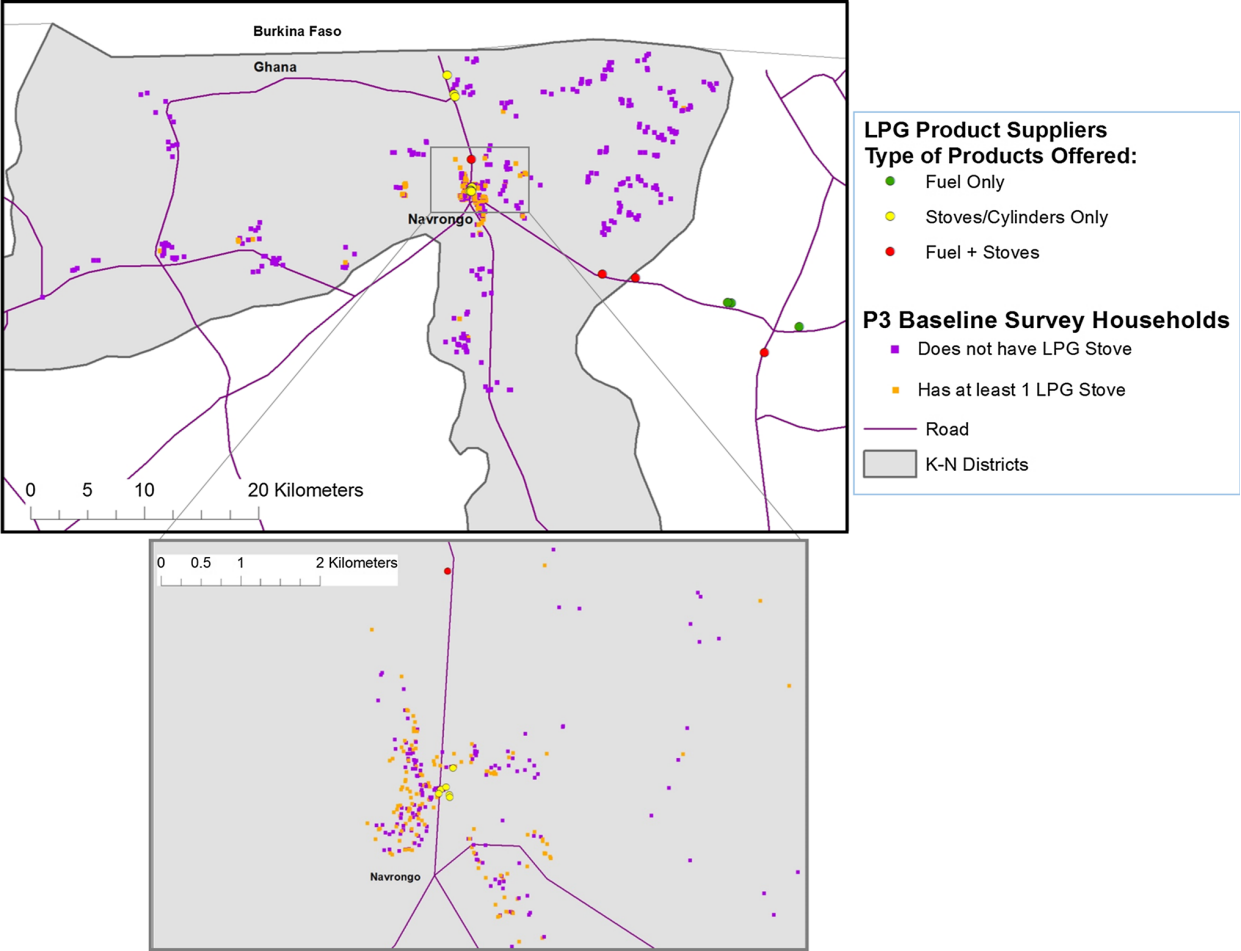
Map of the study area showing locations of LPG suppliers and sampled households.

Figure 1 maps LPG suppliers (by type of products sold), as well as surveyed households (by ownership of LPG stoves). A total of nine retail shops are located across the KND selling LPG stoves and/or cylinders (not fuel) and just one refilling station supplying LPG fuel. Six additional refilling stations are located outside of the KND but in the Upper East region. Two of these appear within the KND border on the map though they are officially outside the district. In total, we interviewed sixteen LPG suppliers (nine retail stove/cylinder shops and seven filling stations).

Out of the 600 recruits, we completed surveys with 592 households (293 rural and 299 urban). As shown in the map, households’ distance to a filling station varies widely within the KND. The majority of households within Navrongo town are within 5 km of the Navrongo filling station, while most rural households are located more than 5 km from an LPG fuel supplier. Ownership of LPG stoves is much higher in the urban sample (51.5%) than in the rural sample (7.5%).

### LPG Supply

#### Characteristics of LPG Suppliers

Table 1 provides background characteristics of LPG suppliers. Out of the 16 LPG supply survey respondents, 6 (37.5%) were the business owners, while 10 (62.5%) were employees. Five respondents (31.25%) were female, but all five of these females were the business owners. The respondents ranged in age from 22 to 59 years (median = 27.5). Eight (50%) of respondents had completed at least tertiary school, six (37.5%) completed secondary school, and two (12.5%) finished primary school. These respondents have been working in this business for a range of 1–27 years (median = 8), with businesses in operation for 1–25 years (median = 6). The number of full-time employees in these businesses ranged from 0 to 5 (median = 1.5).

**Table 1:** Statistics on P3 LPG Supply Survey Respondents and Businesses.

Total number of businesses interviewed	16	
Products provided by business	LPG fuel	7 (43.8%)
	LPG stoves	12 (75.0%)
	LPG cylinders	13(81.3%)
Respondent’s role in business	Owner	6 (37.5%)
	Employee	10 (62.5%)
Respondent’s gender	Male	11 (68.8%)
	Female	5 (31.3%)
Respondent’s age	Min.	22
	Median	27.5
	Max.	59
	Mean	32.1
Respondent’s highest level of education completed	Never attended school	0 (0%)
	Primary	2 (12.5%)
	Secondary	6 (37.5%)
	Tertiary/higher	8 (50.0%)
Number of years respondent has been in this business	Min.	1
	Median	8
	Max.	27
	Mean	8.7
Number of years this business has been in operation	Min.	1
	Median	6
	Max.	25
	Mean	7.5
Number of full-time employees (35 h or more per week)	Min.	0
	Median	1.5
	Max.	5
	Mean	2.1
Own or rent business property	Own	10 (62.5%)
	Rent	6 (37.5%)

#### Types of LPG Products and Pricing

**Figure 2:**
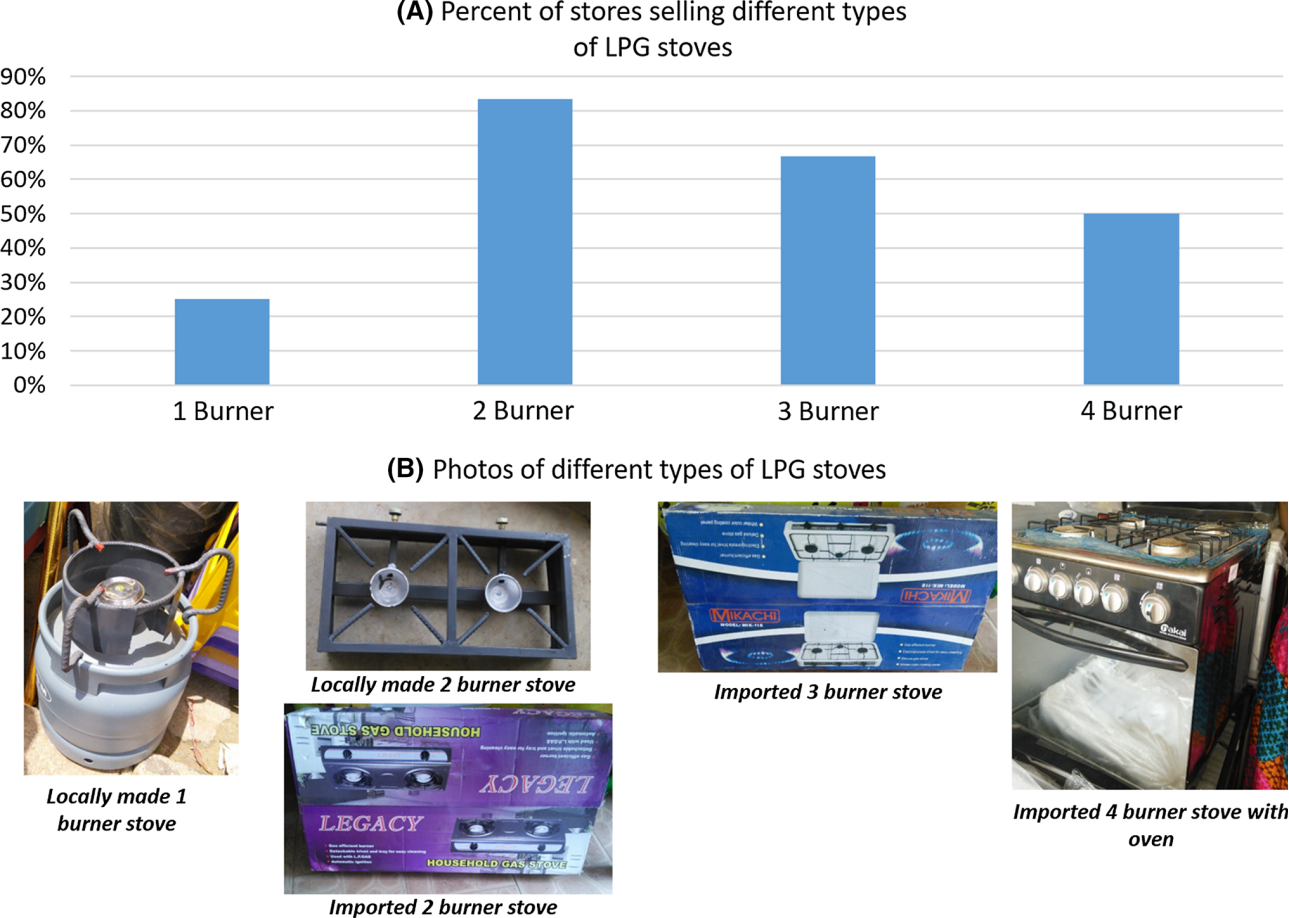
Proportion of stove types sold in businesses carrying LPG stoves, with photographs of typical stove models.

The LPG businesses interviewed sold stoves ranging from a simple one burner model to four burner stoves with ovens. Figure 2 shows the proportion of stores selling each of these stove types, with photographs of typical stove models. The price range for the 1-burner stove was US$3.41 to US$9.10; 2-burner US$13.18 to US$22.73; 3-burner US$15.91 to US$25; and 4 burner US$90.91 to US$159.09. Shop owners reported that the 2-burner stoves were their top sellers.

Cylinder sizes range from 3 to 52 kg. The median price for an empty 3-kg cylinder was US$21.59; 5–8 kg was US$25; and 12–15 kg was US$31.82. LPG refilling station operators gave current LPG prices ranging from US$0.47 to US$0.49 per liter with a median of US$0.48. These operators reported that the price of LPG changes frequently due to deregulation of petroleum products, world market price, and inflation.

#### Supplier Perceptions of Barriers to LPG Adoption

When suppliers were asked open-ended questions about reasons, some urban households had not gotten LPG stoves, cost of stoves, cylinders, and/or fuel was mentioned by 12 out of 16 respondents, while 7 respondents mentioned safety concerns. In addition to these barriers, suppliers also said that rural users faced challenges related to distance to the LPG filling station and lack of information about LPG.

### LPG Demand and Use

#### Background Characteristics of Surveyed Households

Table 2 provides descriptive statistics for the households, including information on stove types and cooking practices. Results indicated that 51.5% of urban households owned an LPG stove, compared to less than 7.5% of rural households. As observed in many studies (Masera et al. 2000, Ruiz-Mercado and Masera 2015), stove and fuel stacking is quite common. In urban areas, 89% of households owned a charcoal stove, and 29.4% had a three-stone stove. About two-thirds of urban households reported using a charcoal stove on the day prior to the survey compared to 27.8% rural households. About 30% of urban households reported using an LPG stove on the previous day compared to 2.4% of rural households. Roughly 20% of urban households reported using a three-stone stove on the previous day compared to 90.1% of rural households. With regard to household characteristics, more than half of the urban primary cooks were also their households’ financial decision makers compared to less than 25% in the rural households. In both urban and rural households over 90% of the primary cook were females. The mean household size was 4.6 (SD = 2.9) in the urban households and 7 (SD = 2.3) in the rural households.

**Table 2:** Descriptive Statistics for Household Sample.

	Rural	Urban	*P* value^a^
# Households	293	299	
*Cooking practices*			
Household has LPG stove	7.5%	51.5%	0.000
Household used LPG stove yesterday	2.4%	29.6%	0.000
Household has charcoal stove	72.0%	89.0%	0.000
Household used charcoal stove yesterday	27.8%	63.6%	0.000
Household has three-stone stove	97.3%	29.4%	0.000
Household used three-stone stove yesterday	90.1%	19.1%	0.000
*Household characteristics*			
Primary cook is financial decision maker	24.6%	50.2%	0.000
Primary cook gender: female	98.3%	91.6%	0.000
Financial decision maker gender: female	4.1%	3.4%	0.111
Primary cook age			
Mean	39.1	42.6	0.002
SD	13.1	13.8	
Primary cook education			
Less than primary	77.8%	36.5%	0.000
Primary/junior high	16.7%	28.1%	
Secondary or higher	5.5%	35.5%	
Household size			
Mean	7.0	4.6	0.000
SD	2.9	2.3	
Household engaged in farming	99.7%	61.5%	0.000
Household has electricity	31.4%	85.6%	0.000
Household has bank account	28.7%	74.6%	0.000
Household has mobile money	36.5%	60.2%	0.000
Household could borrow GHC2000	34.8%	45.5%	0.008
More than half of neighbors have LPG (only asked of urban respondents)	N/A	37.8%	N/A

^a^*P* values are for tests of the null hypothesis that means or frequencies are equal across urban and rural subgroups. These are calculated from *t* tests (for continuous variables) and Chi-squared tests (for binary variables).

#### Predictors of Household Ownership of LPG Stove

**Figure 3:**
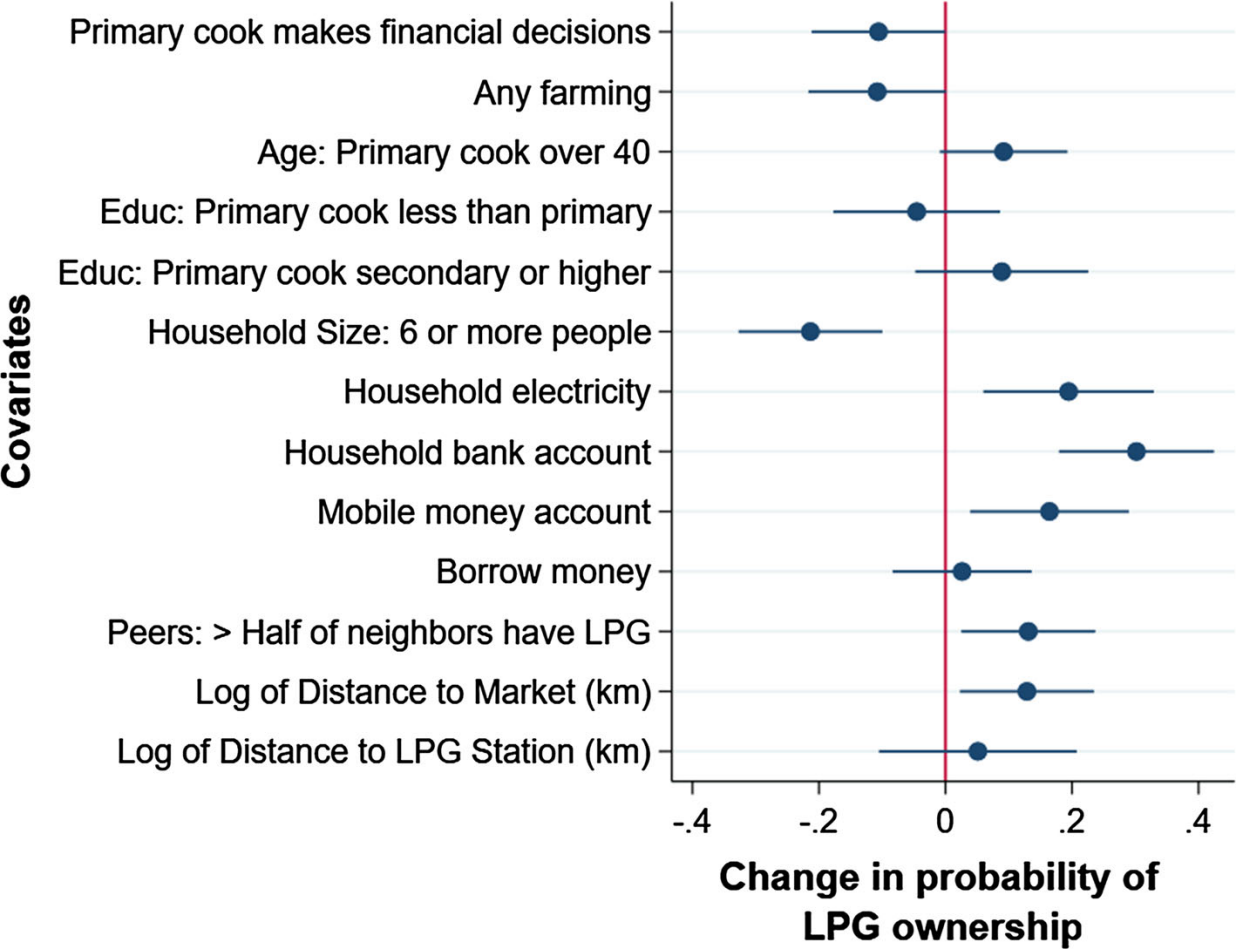
Plot of regression coefficient point estimates (circles) and 95% confidence intervals (lines) from linear probability model for LPG ownership among urban households. *n* = 298 (urban households only), *R*^2^ = 0.35.

Figure 3 plots regression coefficients (point estimates and 95% confidence intervals) derived from a linear probability model examining determinants of LPG ownership. Interestingly, households where the primary cook was also the financial decision maker were less likely to own an LPG stove, as were households involved in farming, while households with older primary cooks (> 40 years) were more likely to own an LPG stove. Education coefficients had the expected signs (negative for cooks with less than a primary education and positive for secondary or higher education, compared to cooks with primary education), but these associations were not statistically significant. Larger households (more than 6 individuals) were less likely to own LPG stoves, while economic well-being (electricity access, bank accounts, and mobile money) was positively associated with LPG ownership. Individuals who reported that more than half of their neighbors had LPG stoves were more likely to own these stoves. Interestingly, households farther from the market were more likely to have LPG stoves, while distance to the LPG filling station had no significant association.

#### Reasons Why Households are not Using LPG

**Figure 4:**
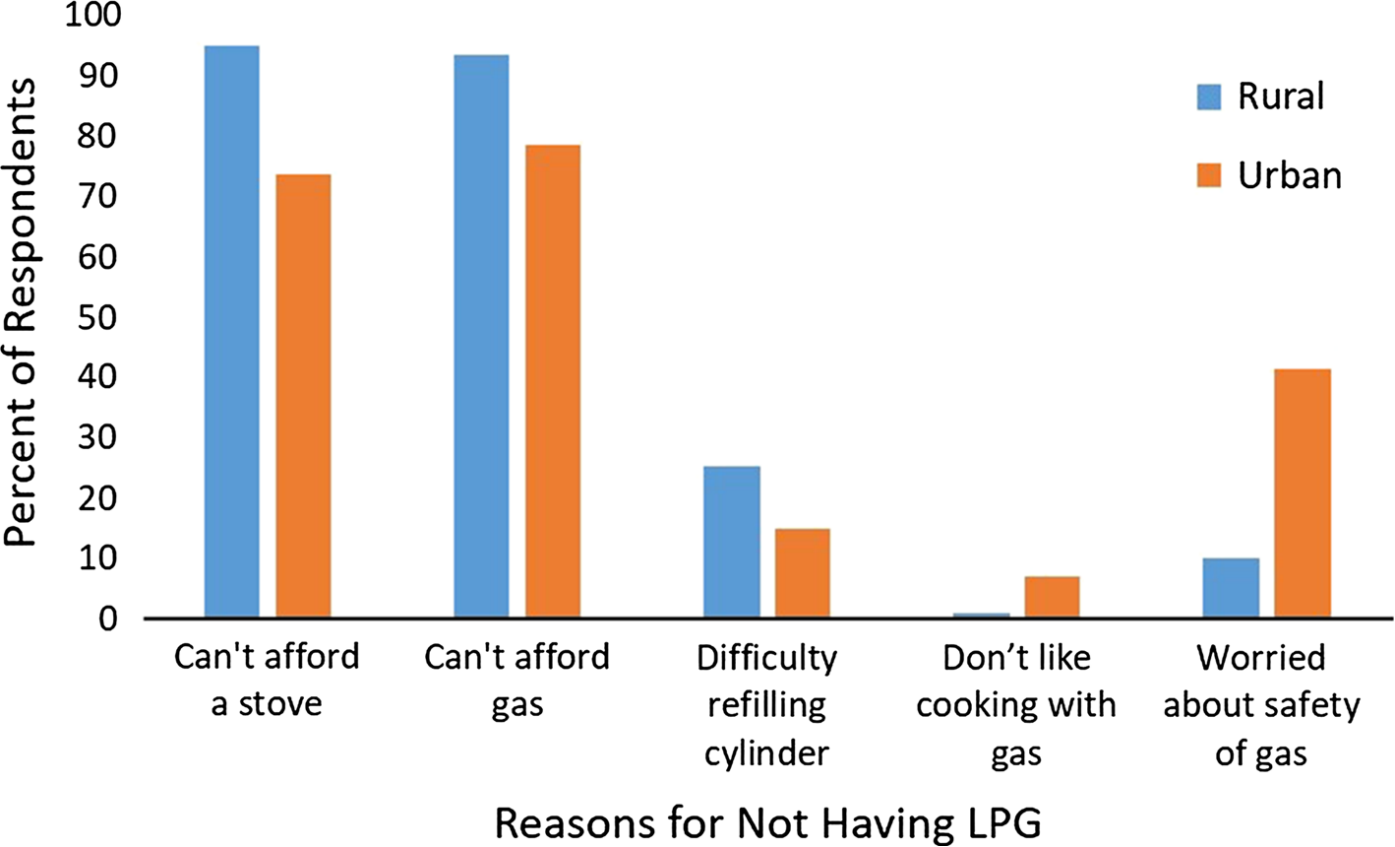
Reasons given by rural and urban household survey respondents for not having an LPG stove. *n* = 412 (269 rural, 143 urban).

Respondents from rural and urban households without LPG stoves were asked why they had not purchased an LPG stove (Fig. 4). Stove and fuel cost were the most commonly mentioned reasons among both urban and rural respondents, though the proportion citing cost barriers was higher in the rural sample. Safety concerns were more commonly mentioned as a reason for not having LPG among urban respondents. In addition, respondents complained about the LPG shortages, not getting the right amount of gas in the cylinder, frequent price increases, and cylinder leakages.

To further probe LPG perceptions, we also asked household respondents whether they agreed, disagreed, or had no opinion about two statements: (1) cooking with gas is dangerous, and (2) cooking with gas shows that a household is wealthy. Overall, most households expressed concerns about the safety of LPG: 85% of rural households and 80% of urban households agreed that cooking with LPG was dangerous. Opinions on whether LPG ownership indicated wealth were more mixed: 77% of rural households agreed compared to 36% of urban households.

#### Stove Features Desired by Households

**Figure 5:**
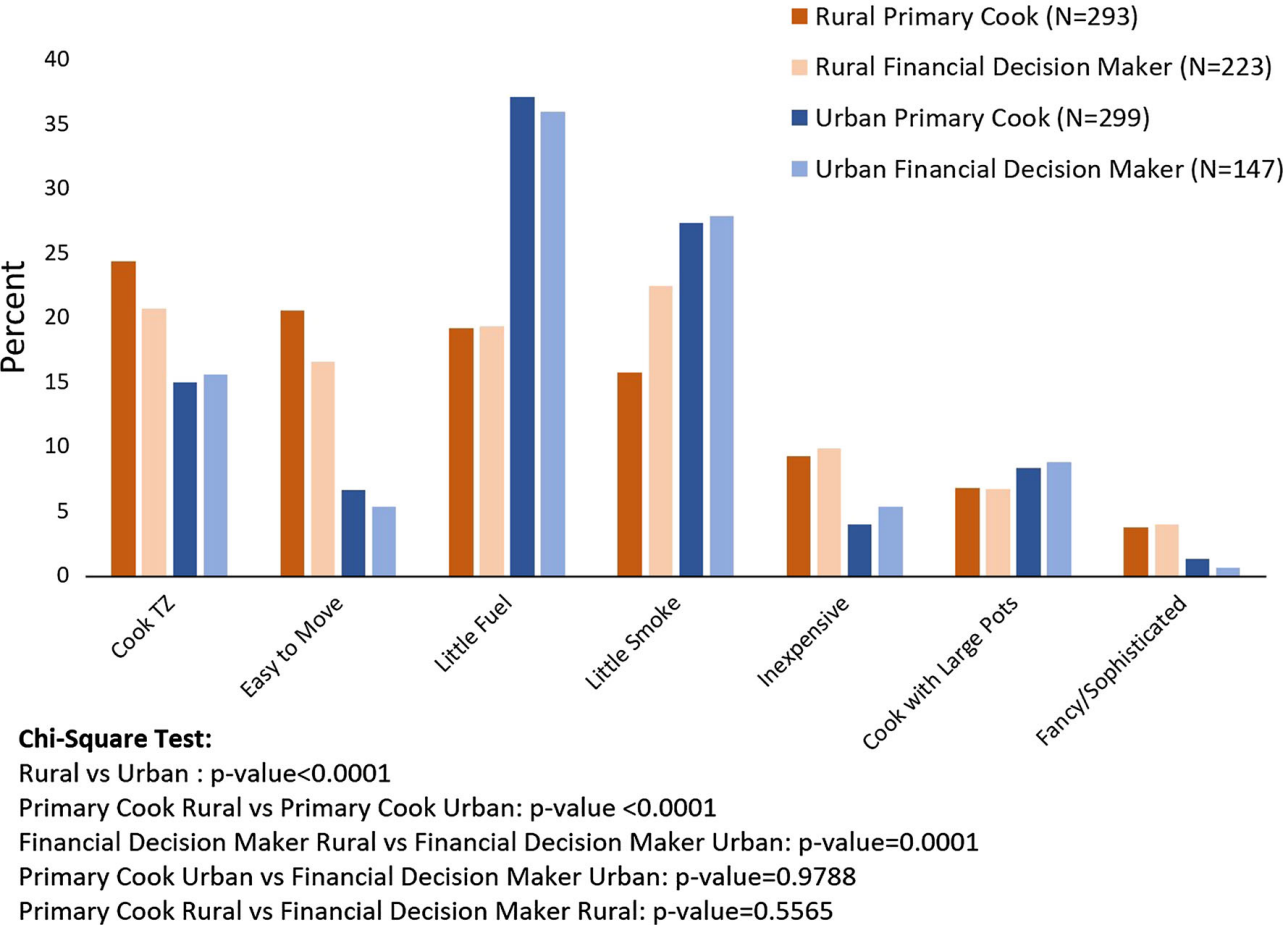
Stove features rated as the most important in the household survey, by respondent type and location.

We asked respondents to select the most important feature that they would consider if they were purchasing a new stove (Fig. 5). Among rural households, primary cooks rated ability to cook the staple porridge Tuo Zaafi (TZ) and ability to move the stove easily as the two most important features, while financial decision makers selected smoke reduction followed by ability to cook TZ as their top two demands. Fuel efficiency ranked third among both primary and financial respondents in the rural areas, while this was the top-rated feature among both types of respondents in the urban sample, followed by smoke reduction. We also asked respondents which type of stove they thought was best for cooking different dishes. For preparing TZ, there was a clear perception that either charcoal (in urban areas) or three-stone stoves (in rural areas) were preferred, with few respondents in either area selecting the LPG stove.

### Policies to Increase LPG Adoption

**Figure 6:**
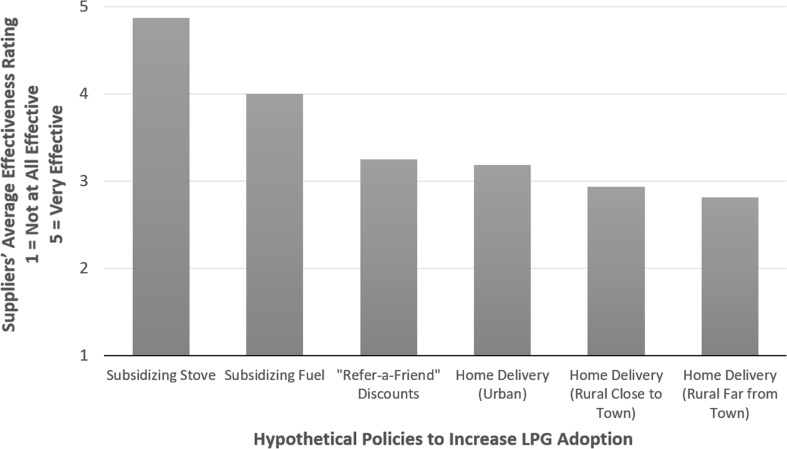
LPG suppliers’ perceived effectiveness of different policy options in encouraging LPG use in the KND. *n* = 16.

**Figure 7:**
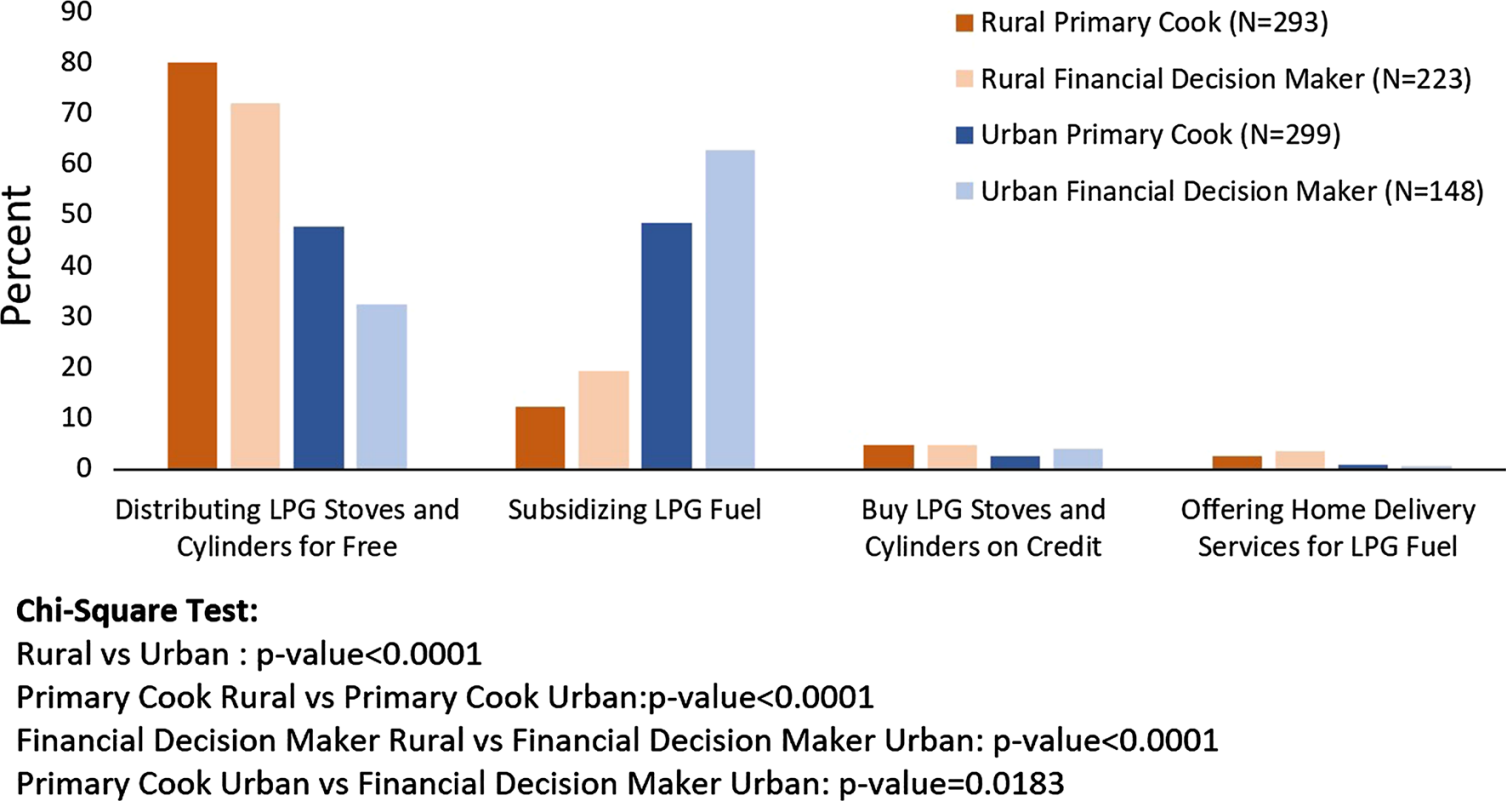
Household survey respondents’ ratings of policy effectiveness, by respondent location. Figure shows the percent of respondents in each subgroup that selected each policy as the most effective policy to increase LPG adoption in the KND.

We asked both suppliers and household respondents to give their opinions on the potential effectiveness of different policies that could be implemented to increase adoption of LPG in the KND. Across the board, subsidies (for hardware and fuel) were perceived as the most effective policies, while other options such as access to credit and home delivery options for LPG fuel were seen as less effective (Figs. 6, 7).

#### Perceptions of Cylinder Recirculation Model

Eleven of the sixteen LPG suppliers had heard of the LPG cylinder recirculation concept, but none knew of any business conducting recirculation. Half of the respondents were in favor of the LPG cylinder recirculation concept. Of those who had previously heard of the LPG cylinder recirculation model, fewer respondents were in favor (45.5%), whereas those who had not previously heard of the model were more supportive (60%). Suggestions provided by respondents to set up the LPG cylinder recirculation model in the KND included having the government provide free LPG cylinders and educating the public on LPG usage, and allaying concerns that “bad cylinders” would be used.

## Discussion

This paper examined both supply- and demand-side factors affecting LPG adoption in the KND. To structure the discussion of the study results, we use the framework of the “4As” of strategic innovation (Anderson and Markides 2007): Awareness, Acceptability, Affordability, and Availability.

### Awareness

Overall, awareness of LPG stoves and fuel was high in both urban and rural areas. All users have some exposure to this technology, but with varying levels of awareness and familiarity. Several rural users reported not knowing how to use LPG as a reason why they had not gotten an LPG stove. Furthermore, widespread safety concerns suggest that increasing awareness of safe ways to use LPG may help increase demand.

Having contact with households that use LPG can increase awareness of this technology, and other studies have found peer effects to be a determinant of LPG adoption (Srinivasan and Carattini 2016). Among urban households, we found that having more neighbors using LPG was associated with higher probability of LPG ownership.

### Availability

Other studies have reported availability and distance to LPG filling stations as factors that hinder LPG adoption and use (Oteh et al. 2015; Srinivasan and Carattini 2016). Within the urban area of the KND, LPG stoves and cylinders were readily available, and our regression results showed that distance to the filling station was not a significant barrier to owning an LPG stove. One possible explanation, based on our observations from the region, is that “suburban” homes on the periphery of Navrongo are larger and homeowners may be somewhat wealthier, such that accessing fuel is not a significant barrier to adoption. Availability is likely to be a bigger barrier for rural households, many of whom are located quite far from retail shops and filling stations and may lack transportation options.

Meanwhile, filling station operators and households both reported occasional shortages of LPG, such that availability may be interrupted periodically in this area.

### Affordability

Affordability was perceived as the main reason for low usage of LPG in the KND. Based on reported prices, the cost of entry for a new LPG user is roughly US$63 (2 burner stove plus 15-kg cylinder plus first refill). This cost is roughly the same as the national average monthly wage of about US$64, and poverty levels are higher in the Upper East region (Ghana Statistical Service 2014). In rural areas, most households are subsistence farmers without a regular source of income. Previous studies have also reported that household income and LPG prices were key determinants of a households decision to use LPG (Kojima 2011; Cecelski and Matinga 2014; Oteh et al. 2015; Vahlne 2017).

In order to improve LPG usage in Ghana and achieve the 50% LPG access target, subsidies for stoves, cylinders, and fuel may be required, particularly in the rural areas. Currently, the main approach of the RLP is to provide free stoves and cylinders to beneficiary households, while requiring payment for LPG fuel. However, a recent evaluation in Nkoranza North District indicated that only 8% of households were using their LPG stoves 18 months post-distribution, due to difficulty accessing LPG filling stations and affordability of LPG fuel (Asante et al. in preparation). Thus, hardware subsidies on their own may not be sufficient to generate widespread adoption and use of LPG.

Other studies have also found that providing access to credit and/or allowing households to pay for stoves in smaller increments over time can be an effective way to increase demand (Beltramo et al. 2015). Interestingly, household respondents did not rate these options very highly, perhaps because they preferred the policy options that reduced the overall cost of LPG stove packages.

### Acceptability

The fact that more than half of urban households already own and use LPG stoves indicates that many users perceive this as an acceptable, desirable technology option. Across rural and urban samples, respondents also expressed a strong preference for cooking options that reduce smoke exposure.

At the same time, our results indicate widespread concerns about the safety of LPG for cooking, consistent with studies conducted in other contexts (Cecelski and Matinga 2014; Oteh et al. 2015). An explosion at a natural gas station in Accra in October of 2017 is likely to exacerbate these fears, though this accident was not directly linked to households’ use of LPG for cooking. Most of the risks for household use of LPG are related to use of old cylinders and cylinder leakages. Cylinder recirculation can avoid some of these problems since households do not have an incentive to continue using older cylinders.

Another acceptability issue involves perceived suitability of LPG stoves for cooking common dishes, particularly TZ. Other work has shown that urban households in this area nearly always cooked this dish over charcoal stoves, while rural households used three-stone fires; LPG was rarely used to prepare this dish (Wiedinmyer et al. 2017).

## Conclusion

Increased adoption of LPG has potential health and environmental benefits in low–middle-income countries (LMICs) (Rosenthal et al. 2018). However, LPG requires a more sophisticated supply infrastructure than previously promoted improved charcoal- and wood-burning stoves, which rely on locally produced or collected fuels. Expansion of this infrastructure from urban to rural areas is thus key to increasing household LPG adoption. This study examined the features of such infrastructure in the KND, which is similar to nascent LPG markets in many LMIC urban and rural areas.

We find several reasons to be optimistic about the potential for scaling up access to LPG in Ghana and similar environments. The national government has made LPG expansion a priority and has been developing national strategies with the support of international institutions such as the Global LPG Partnership.

To further expand service to—and increase demand among—households in this region, we offer two main policy recommendations. First, the push toward cylinder recirculation should be accelerated. This policy has the potential to make LPG use safer, as well as facilitating larger spatial distribution to reach rural customers. Public education emphasizing smoke reduction and potential health benefits of LPG, along with guidelines for safe use and the role of recirculation in addressing safety concerns, should accompany this policy implementation.

Second, attempts to expand LPG use in this area will need to grapple with affordability challenges inherent in areas with high poverty rates and cash constraints. Even when stoves are provided for free, difficulties accessing and paying for fuel can limit LPG uptake. Public–private partnerships that can provide access to credit, as well as targeted fuel subsidies for poorer households, may need to be incorporated. Ghanaians should continue to share lessons with other countries and international organizations pushing for cleaner cooking solutions.
